# Electronic Medical Records implementation in hospital: An empirical investigation of individual and organizational determinants

**DOI:** 10.1371/journal.pone.0234108

**Published:** 2020-06-04

**Authors:** Anna De Benedictis, Emanuele Lettieri, Luca Gastaldi, Cristina Masella, Alessia Urgu, Daniela Tartaglini

**Affiliations:** 1 Department of Healthcare Professions, University Hospital Campus Bio-Medico, Rome, Italy; 2 Faculty of Medicine & Surgery, University Campus Bio-Medico, Rome, Italy; 3 Department of Economics, Management and Industrial Engineering, Politecnico of Milan, Milan, Italy; University of Milan, ITALY

## Abstract

The implementation of hospital-wide Electronic Medical Records (EMRs) is still an unsolved quest for many hospital managers. EMRs have long been considered a key factor for improving healthcare quality and safety, reducing adverse events for patients, decreasing costs, optimizing processes, improving clinical research and obtaining best clinical performances. However, hospitals continue to experience resistance from professionals to accepting EMRs. This study combines institutional and individual factors to explain which determinants can trigger or inhibit the EMRs implementation in hospitals, and which variables managers can exploit to guide professionals’ behaviours. Data have been collected through a survey administered to physicians and nurses in an Italian University Hospital in Rome. A total of 114 high-quality responses had been received. Results show that both, physicians and nurses, expect many benefits from the use of EMRs. In particular, it is believed that the EMRs will have a positive impact on quality, efficiency and effectiveness of care; handover communication between healthcare workers; teaching, tutoring and research activities; greater control of your own business. Moreover, data show an interplay between individual and institutional determinants: normative factors directly affect perceived usefulness (C = 0.30 **), perceived ease of use (C = 0.26 **) and intention to use EMRs (C = 0.33 **), regulative factors affect the intention to use EMRs (C = -0.21 **), and perceived usefulness directly affect the intention to use EMRs (C = 0.33 **). The analysis carried out shows that the key determinants of the intention to use EMRs are the normative ones (peer influence) and the individual ones (perceived usefulness), and that perceived usefulness works also as a mediator between normative factors and intention to use EMRs. Therefore, Management can leverage on power users to motivate, generate and manage change.

## Introduction

Healthcare is the most complex and fast-moving industry that exists. New digital technologies are constantly being developed, all with the potential to support the clinical practice by bringing many advantages into the healthcare sector [1]. Nevertheless, the healthcare industry has lagged behind other sectors in the adoption of Information Technology (IT) in the workplace [2]. Electronic Medical Records (EMRs) have long been considered a key factor for improving healthcare quality and safety, reducing adverse events for patients, decreasing costs, optimizing processes, improving clinical research and obtaining best clinical performance [e.g., 3–5]. However, the pace of adoption of EMRs–as other digital technologies–in healthcare continues to lag [2,6], and hospitals continue to experience resistance from professionals to accepting digital technology [7]. Though many research and development programs exist and venture capital investment has been growing, successful IT projects in healthcare continue to be rare, and a plan to accelerate innovation is needed beginning with a diagnosis of the problem [2]. Some studies analyzed both individual and organizational factors that affect the acceptance and implementation of technology [8], but they have generated mixed results [9]. Indeed, mechanisms that drive the adoption and implementation of IT in hospitals remain unclear. Organizational studies conceive organizations as strongly institutionalized settings in which individual behaviours are influenced by regulations, social norms and cultural systems [10,11]. In contrast, Information Science has mostly adopted user acceptance models, which emphasise individuals’ rational and volitional assessment of the costs and benefits they would attain from the new digital technology [11].

Hospitals are highly institutionalized and regulated contexts, in terms of regulatory oversight and professional roles, and are operationally and technically complex [12]. Physicians and nurses have a high level of professionalism and they often affiliate within their specialities via professional training and participation in speciality-focused organizations [13]. Successful adoption or perceived usefulness of EMRs by others within their specialities may influence hospital professionals’ decisions, particularly if they are uncertain about individual benefits. Nevertheless, the majority of academic research in IT adoption in healthcare focused on the individual level [14]. The most widely used model to explore issues related to the acceptance of technology is the Technology Acceptance Model (TAM) [15], which identifies two main antecedents the perceived usefulness and the perceived ease of use of technology. The TAM has been validated in multiple settings [e.g. 16–18]. In its basic framework, the end user’s attitudes and perceptions regarding the use of new technology determine the user’s behavioural intention to use it. Institutional theory, instead, is based on the assumption that individual behaviours are modelled by regulations, social norms and meaning systems and that institutions embodied in routines rely on automatic cognition and uncritical processing of existing schemata and privilege consistency with stereotypes and speed over accuracy [19]. Thus, in this theory, normative and cultural conditions are co-determinants of the adoption of new technologies [20]. The use of institutional theory in Information Science is rare compared to other fields such as organization science [21]. However, several studies have used an institutional approach for exploring the adoption of technology considering institutional forces as crucial to shaping organizational actions and the opinions of the decision-makers [22,23,24].

Both institutional theory and user acceptance models have independently tried to incorporate elements of the other theory to enrich their explanatory power [2]. User acceptance models have incorporated the direct effects of social influences and organizational conditions on individuals’ behavioural intention [25,26], and institutional studies have demonstrated that even when professionals are subject to institutional influences, their self-determination plays an important role even in highly-institutionalized and regulated settings such as hospitals [27]. Previous studies about technology acceptance and adoption compared individual and social levels including environmental factors [22,28–30], typically based on the diffusion of innovation theory (DOI) [31] or the TOE (technology, organization, and environment) framework [32]. Moreover, only a few studies have tested both explanations (institutional and individual) in an integrative framework [23] to explain the behaviour of organizations.

The main purpose of this study was to explore which are the main determinants of hospital professionals’ intention to use EMRs through a novel theoretical model that combines organizational theories and technology acceptance models. By combining these theories, this study investigated the interplay between organizational and individual factors, thus offering novel insights on the determinants of hospital professionals’ acceptance of digital technology by showing how and to what extent the interplay between individual and organizational determinants might trigger or inhibit the acceptance of digital technology. This study focused on perceived usefulness and perceived ease of use as explanatory factors at the individual level, and on inter-hospital normative and regulative forces as explanatory factors at the organizational level. Intention to use has been preferred to repetitive use as the dependent variable. This choice is because of the still relatively low adoption rate of EMRs in many Countries such as Italy, where this study is located. In the specific case of Italy, a recent report issued by the Politecnico di Milano within the research activities of the Permanent Observatory of Digital Transformation in Health Care [33] pointed out that only 53% of Italian hospitals have in place an EMRs for therapy management, only 30% of Italian hospitals have in place an EMRs that collects vital parameters and informed consensus, and only 19% of Italian hospitals have in place an EMRs that supports clinical decision-making. In this view, a large number of Italian hospitals–as well as hospitals from other Countries who are still lagging in the adoption of EMRs–is expected to commit in the next years to adopt EMRs and the understanding of which individual or organizational factors might shape hospital professionals’ intention to use EMRs might contribute to the successful adoption and implementation of such and other digital technologies. In this view, the results of this study might be valuable for hospital managers and professionals of different countries who are going to invest in the digital transformation of their hospitals.

## Material and methods

### Ethics statement

The study has been approved by the Ethics Board of the University Hospital Campus Bio-Medico of Rome. (Approval number: 61/16 OSS ComEt CBM), and written consent has been obtained by professionals involved in the study.

### Theoretical background

**Fig 1:**
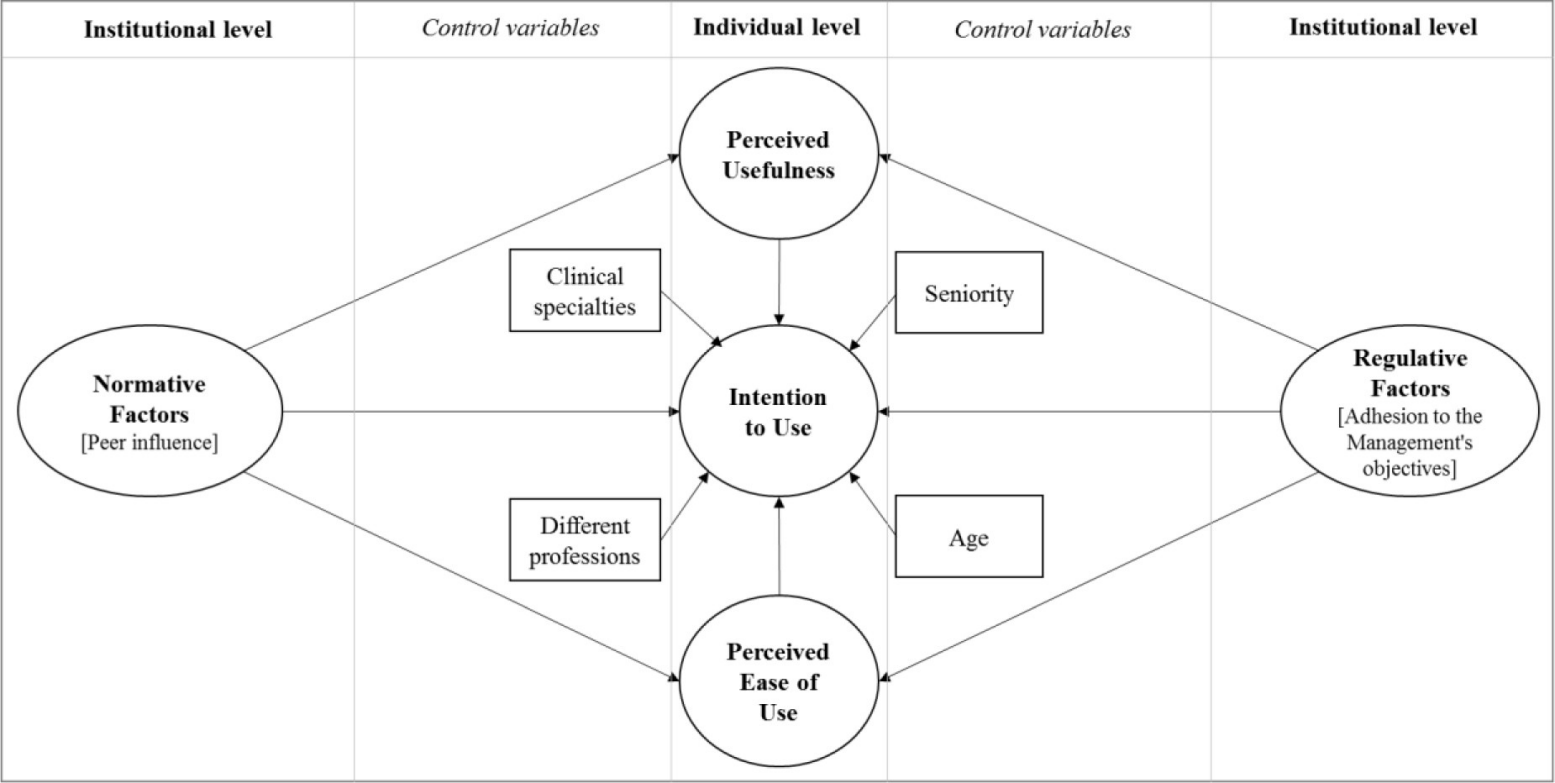
Research framework.

To evaluate the potential interplay between individual and institutional variables, a research framework has been created (Fig 1). The framework integrates into a coherent view of two theories that belong to two different bodies of literature:

- The Technology Acceptance Model (TAM), from Information Science, that has been widely used in the last decades in healthcare to understand what leads professionals or patients to accept or reject Information Technology [15];
- The Institutional Theory, from Public Management, that has been largely adopted in the last decades to assess how institutional factors shape professionals’ behaviours [34–36].

#### Technology acceptance model

Davis introduced the TAM in 1989 [15]. The main problem raised by the author was to understand what leads people to accept or reject Information Technology. In this regards, two main variables have been identified: the perceived usefulness and the ease of use. Perceived usefulness measures “the degree to which a person believes that using a particular system would enhance his or her job performance” [15], and therefore induces individuals to use technology as it allows to obtain better results. On the other hand, the ease of use measures “the degree to which a person believes that using a system would be free of effort” [15] and induces the potential users to use a certain technology since it requires low energy expenditure while it may bring advantages. The first one induces an individual to use technology as it allows to obtain better results in his work; the ease of use, on the other hand, stimulates potential users to use a certain technology since many advantages are supported with low energy expenditure.

#### Institutional theory

The Institutional Theory refers to a line of organizational research that recognize the significant organizational effects that are associated with the increase of cultural and social forces. According to Scott [34–36], “Institutions are made up of cultural-cognitive, normative and regulative elements, which together with associated activities and resources offer stability and meaning to social life.” These three forces are present in totally developed institutional systems, with economists and political scientists emphasizing regulative, sociological and normative factors, and anthropologists and organizational theorists emphasizing cognitive-cultural factors. According to this perspective, individuals are embedded in institutional pillars that limit the scope of their rational assessment and direct the engagement of specific behaviours [34–36]. Scott [34–36] defines the three institutional pillars as follows:

- *regulative pillars*: which regard the existence of regulations, rules and processes whose breach is monitored and sanctioned;
- *normative pillars*: which introduce a social dimension of appropriate behaviours in the organization;
- *cultural pillars*: which emphasize the use of common schemas, frames, and other shared symbolic representations that create an attachment to the ‘appropriate’ behaviour.

### Research framework

Consistently to our research questions, we combined the two theories described above to develop an original, comprehensive research framework where individual and institutional determinants have been interlinked to explore their potential interplay in explaining hospital professionals’ intention to use an EMR. Coherently to past researches about user acceptance of new technologies [36,37], we considered age and job seniority as key control variables. Additionally, to narrow the knowledge gap about how hospital professionals belonging to either different profession (e.g., physicians vs. nurses) or different speciality (e.g., cardiology vs. orthopaedics) might be interested to use an EMR, we included clinical speciality and profession as control variables. Fig 1 offers a synoptic view of our research framework, where the independent variable (i.e., the intention to use an EMR) is explained by individual factors from TAM (i.e., perceived usefulness and perceived ease of use) as well as by institutional factors from Institutional Theory (i.e., regulative factors that refer to the degree of adhesion to hospital managers’ goals, and normative factors that explain the peer influence among hospital colleagues. Control variables have been also displayed.

According to the research questions and the research framework, the following research hypotheses (H) were stated: H1: Individual factors (perceived usefulness, perceived ease of use) directly affect the intention to use EMRs; H2: Organizational factors (normative and regulative factors) directly affect individual factors and the intention to use EMRs; H3: Some control variables (age, seniority, clinical specialities and different professions) directly affect individual factors and the intention to use EMRs.

### Setting and research methodology

Given the explorative nature of this study, a single case study research design has been adopted. The choice of a single case study offers the opportunity to eliminate potential confounding factors due to the heterogeneity–in terms of strategy, legacy, professionals’ behaviours and technology infrastructure–that different hospitals might show. We selected the Teaching Hospital Campus Bio-Medico (CBM) in Rome (Italy) as an adequate setting for investigating our research questions. This hospital is mid-size (around 300 beds), many-disciplines, teaching and private. Being a teaching hospital, there is more room for divergent goals between professionals and managers, thus creating the correct setting where to investigate the interplay between individual and organizational factors. Being many-discipline, there is room to study the potential conflict among professionals from different disciplines concerning the intention to use EMRs. Finally, being mid-size, CBM is a valid setting to observe the potential divergence between nurses and doctors in the intention to use EMRs. A quantitative study has been performed using a survey administered to hospital professionals (physicians and nurses). The questionnaire has been designed based on the scales identified in the literature and reviewed in detail by the authors. Moreover, a pilot test of the questionnaire has been carried out before the survey. The initial questionnaire comprised 20 items that were reviewed for face validity by a panel of four experts, consisting of one nurse and one physician—with more than 9 years of work experience -, and two engineers with expertise in Information Science. Panel members were asked to evaluate each statement for clarity, ease of use and appropriateness. Based on their comments and suggestions, five items were removed and changes were made in the wording of several items to increase clarity.

This 15-item questionnaire was tested for content validity by 10 experts not involved in the preceding phase to identify its ability to measure the determinants of the intention to use EMRs in hospitals and to identify, for each item, utility, consistency with the research objectives, easy of reply and other important aspects to take into account. Audio-recorded individual interviews using a semi-structured grid were carried out with 10 experts including two nurses, three head nurses, two managers and three physicians. The interviews lasted 60 minutes on average and were conducted in a designated room by three researchers: one acted as the interviewer, the other two helped with audio-recording and with filling out the grid for item evaluation. Based on the expert evaluation, three items were modified.

The questionnaire consists of two main sections: scales and constructs of the proposed model; control variables and characteristics of respondents. Eleven items evaluated individual variables, in particular, the scale for the measurement of perceived usefulness has been adapted from the studies of Venkatesh [38,39]. Organizational variables were explored through 4 items related to normative and regulative factors. The scale for the measurement of normative and regulative factors has been adapted from the study of Scott [20]. The survey items are available in Annex (S1 Table). Additional questions have been designed to gather demographic and sample information. All questionnaire items related to the constructs of the proposed model were explored using a 7 point Likert scale with 1 indicating “strongly disagree” and 7 “strongly agree”. The first re-call has been made one week after the expiration date for compilation. Three days after the first follow-up, the second recall has been sent. Finally, three days after, the third recall has been sent.

The statistical analysis was performed using the software Stata 14.1®. The internal consistency was evaluated through Cronbach’s Alpha coefficients, the path analysis was performed to test the proposed model considering a p-value of <0.05 as significant. The correlation between profession (doctors vs. nurses) and the answers provided for each item was analyzed through the Fisher’s test; a p-value of <0.05 was considered significant.

The study has been approved by the General Management and the Ethics Board of CBM. The link for the online questionnaire was sent by e-mail to 380 nurses and 250 physician representatives of different clinical areas. All questionnaires were filled out anonymously in a period between February and September 2018. The final sample included 114 hospital professionals (response rate 19%), composed by 78 (68%) nurses and 36 (32%) physicians. They were 84 (74%) females and 30 (36%) males, aged 37.4 years on average (range 23–66, SD 9.6), with a mean work experience of 13.24 (range 0.5–41, SD 8.73). The sample of respondents has been compared–in terms of age, gender and clinical experience–to the whole population of doctors and nurses enrolled at CBM confirming the absence of potential response biases related to the non-respondents.

## Results

### Questionnaire’s internal consistency

The internal consistency of constructs was evaluated through Cronbach's Alpha coefficients, values greater than or equal to 0.7 were considered acceptable. (α ≥ 0.90 were considered excellent; 0.8 ≤ α < 0.9 good; 0.7 ≤ α < 0.8 acceptable; 0.6 ≤ α < 0.7 questionable; 0.5 ≤ α < 0.6 poor; α < 0.5 unacceptable) (Table 1).

**Table 1:** Measurement properties of constructs.

Construct	Items (corresponding to the survey questions)*	Cronbach's alpha
***Perceived Usefulness***	**A**. I’m convinced that the EMR will help me carry out my tasks faster.	0.79
	**B**. Using the EMR will greatly improve the effectiveness of my work.	
	**C**. Using the EMR in my work will greatly increase my productivity.	
***Perceived Ease of Use***	**A**. The use of EMR will increase my workload.	0.73
	**B**. Using the EMR I will have more control of my work.	
	**C**. I will have problems to use the EMR.	
	**D**. I will be able to get the system to do what I want.	
	**E**. The EMR will be easy to use.	
***Intention to Use***	**A**. If I had the opportunity I would use the EMR,	0.76
	**B**. If I had the opportunity I would use the EMR for most of my work’s processes.	
	**C**. If I had the opportunity I would work in a Hospital where the EMR is already used.	
***Peer Influence (Normative pillar)***	**A**. The colleagues I value most believe that I should systematically use the EMR.	0.82
	**B**. The colleagues I value most consider the use of EMR as essential for the Hospital.	
***Adhesion to Management’s objectives (Regulative pillar)***	**A**. I very much agree with most of the objectives of the management.	0.77
	**B**. I often come into conflict with the management on the priorities to give to my work (reversed).	

*All items were measured on a 7-point Likert scale, where 1 = strongly disagree, 2 = moderately disagree, 3 = somewhat disagree, 4 = neutral (neither disagree nor agree), 5 = somewhat agree, 6 = moderately agree, and 7 = strongly agree.

### Determinants of current behaviours

**Fig 2:**
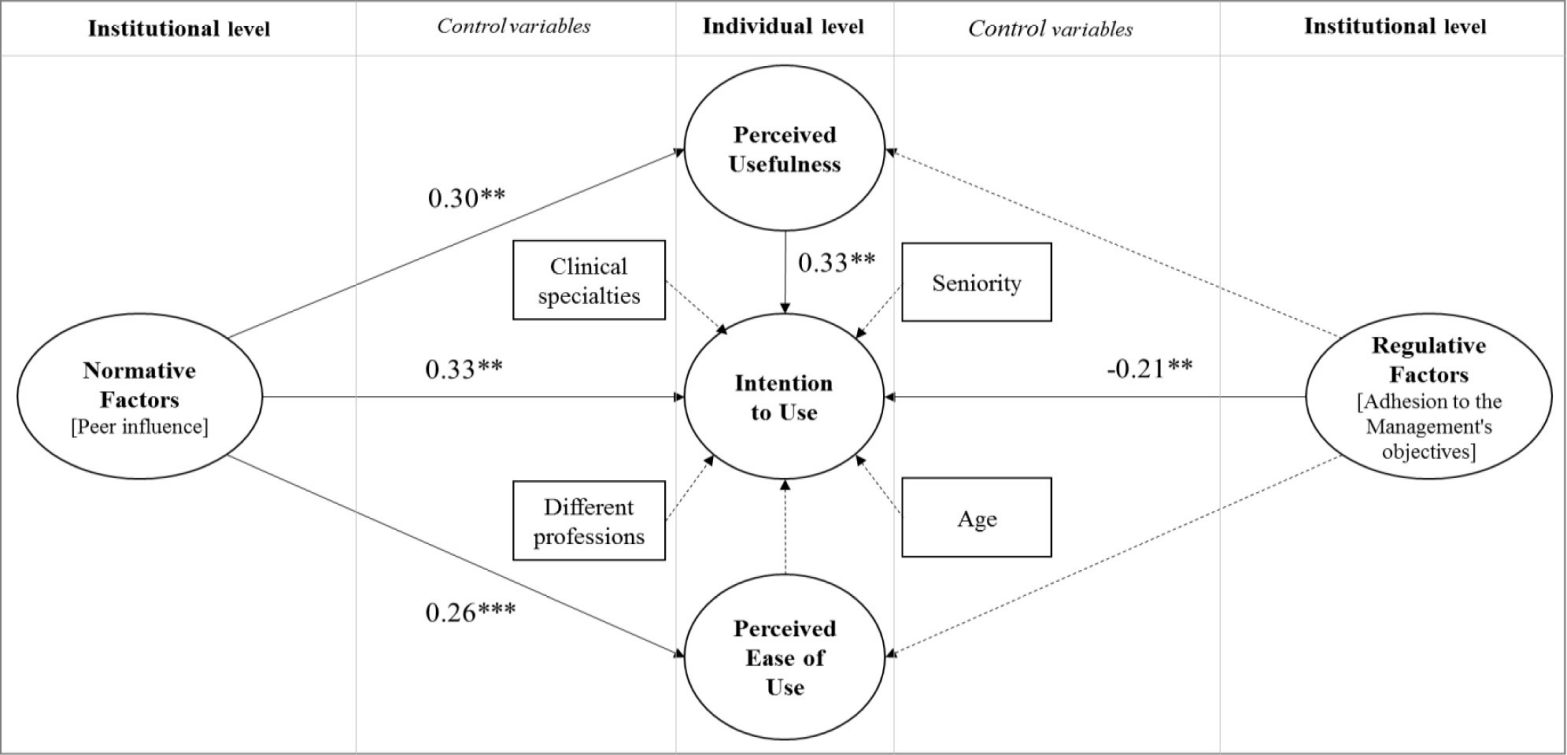
Determinants of current behaviours.

Data show that both physicians and nurses expect many benefits from the use of EMRs. In particular, they think EMRs will have a positive impact on relevant factors such as quality, efficiency and effectiveness of care; handover communication among healthcare workers; teaching, tutoring and research activities; greater control of their tasks. Data confirm that perceived usefulness (C = 0.33**) directly affects the intention to use EMRs. Concerning the organizational factors, data prove that there does exist an interplay between them and individual determinants. In fact, normative factors directly affect perceived usefulness (C = 0.30**), perceived ease of use (C = 0.26**) and intention to use EMRs (C = 0.33**). Regulative factors affect the intention to use EMRs, with a negative sign (C = -0.21**). Control variables (i.e., age, seniority, clinical area and profession) have no impact on other variables in our model. Fig 2 offers a graphical representation of our results.

Moreover, the findings show a significant correlation between being a nurse or a physician and the perceived ease of use and intention to use EMRs. In particular, more nurse than physicians perceive EMRs as easy to use (p = 0.019 for the item “the EMR will be easy to use”) and state that they would like to use it (p = 0.01 for the item “if I had the opportunity I would use the EMR for most of my work’s processes”).

## Discussion

This study sought to better clarify the relationship between organizational and individual determinants of the intention to use EMRs in a hospital setting by nurses and physicians. Previous studies [40–46] have focused mainly on either the barriers or the facilitators that might impact on the implementation of EMRs, but, to the best of authors’ knowledge, it has never been deepened if and how organizational and individual factors do interact and affect jointly hospital professionals’ motivation to use EMRs. Findings confirmed the positive role played by the perceived usefulness as driving individual factor to the intention to use EMRs and shed light on the significant positive role played by the normative (peer influence) factors [2], both with direct and indirect effects. In this view, hospital managers can leverage on lead peer influence (i.e., innovation champions) to motivate, generate and manage change and generate a virtuous circle inside the hospital to motivate the use of EMRs. The EMRs implementation process should take into account that professionals need proper time to re-establish control over their tasks and processes. The introduction of EMRs in daily clinical practice changes the status quo and, if, on one hand, it allows many new opportunities, on the other hand, it involves changes that can have different effects on hospital professionals also based on their own characteristics, knowledge, skills and work type. In general, this is what happens in the case of effective implementation, while the consequences of poorly managed implementation can be very complex and involve a greater expenditure of time, energy and money to restart the processes at the previous speed and functionality. In this sense, to increase the motivation of users in all phases of the project represent an essential point for effective management of change. This study confirms the importance of involving front-line professionals, as soon as the hospital decides to start the implementation phase to increase their motivation to use EMRs. In fact, as a result of their involvement, professionals will better understand the rationale of this technological shift and their perception of usefulness will increase consequently. Moreover, it is important to consider that, as reported by Gastaldi et al. [2] in the absence of coercive mechanisms, institutional pressures toward EMR use are primarily normative and/or mimetic [2].

In the study, the construct “Regulative factor” has been derived from the Institutional theory and is aimed at exploring the pressure that a hospital professional might perceive from the goals set by hospital managers. This pressure is intended to be independent of the specific strategy/initiative and to be a general availability of a hospital professional to align his/her behaviour to the goals set by hospital managers. An example of a question is: “I very much agree with most of the objectives of the management”. The regulative factor should be analyzed together with the construct “Normative factor” that crystallizes the perceived pressure from peers. Hospitals are intended as professional bureaucracies where professionals feel more the pressures from peers rather than from apex managers. What is interesting is that the regulative factor affects negatively the intention to use, meaning that more the general agreement with managers’ goals less the intention to use an EMR. This finding might appear as counter-intuitive and contrary to what has been found in other studies [47]. This result cannot be explained by the potential misalignment between hospitals managers’ goals and those of physicians and nurses, being the former more focused on the efficiency and the latter on the effectiveness of care delivery. Managers at CBM have proved to be committed to the quality of care and not to efficiency strategies that might reduce the effectiveness of care. This context is quite typical in Italy, where the tensions between “medicine” and “management” are less evident than in other countries, such as in the US. We think that the negative impact of the regulative factors on the perception of usefulness is because hospital managers did not detail enough their goals about the digital transformation of care delivery, thus impacting negatively on hospital professionals’ perception about the usefulness of an EMR. Being these goals enough general–e.g., providing support to research activities and care delivery, promoting efficiency and process redesign–while the linkage between the regulative factors and the perception of usefulness failed to materialize, the linkage between the regulative factors and the intention to use EMRs became negative as hospital professionals lost the connection between EMR usage and managers’ goals. In this view, more contextualized goals about the usage of EMR are expected to positively affect the intention to use it among those professionals who are more willing to be adherent to managers’ goals. This finding should be tested and confirmed by further replication studies that might capture more in detail the relationships between regulative factors and either the perceived usefulness or the intention to use. For instance, it might be valuable to understand whether and how the co-development of hospitals goals between managers and professionals might impact these relationships as well as the specific content of hospital goals (financial vs. quality of care, operative vs. research).

## Conclusion

This study offers original insights to further the ongoing debate about the digital transformation of hospitals, with a focus to EMRs. Our results show that there is an interplay between individual and organizational factors in shaping hospital professionals’ intention to use EMRs. The study showed that the main determinants of the intention to use EMRs are the normative ones (peer influence) and the individual ones (perceive usefulness).

From an academic viewpoint, the study offers an original perspective and a new theoretical framework, which combines organizational theories and technology acceptance models to explain hospital professionals’ acceptance of EMRs. In particular, the results confirm the importance of individual variables, not only as directly related to the acceptance of new technology, but also as important mediators between institutional variables and acceptance, thus highlight and confirming the importance of the connections between organizational studies and information science.

Despite the original contributions, this study suffers at least two limitations that should be addressed by future research. First, the research design is based on a single case study. Further research should consider a multi-centre design, thus allowing the generalization of our results. Moreover, a multi-centre study will allow exploring the role that hospital characteristics–in terms of strategy, legacy, etc.–might have on shaping both the organizational and individual factors investigated in this study. Second, this study investigated the intention to use EMRs as the dependent variable. Further research should consider hospitals where EMRs are already mature technologies, thus allowing the investigation of the actual use and which factors might facilitate/inhibit the translation of the intention to use into actual use.

## Supporting information

S1 Table Questionnaire. (DOCX)

S2 Table Perceived usefulness. (DOCX)

S3 Table Perceived ease of use. (DOCX)

S4 Table Intention to use. (DOCX)

S5 Table Normative factors (Peer influence). (DOCX)

S6 Table Regulative factors (Adhesion to the management objectives). (DOCX)
