# Supplementary material for: Electronic Medical Records implementation in hospital: An empirical investigation of individual and organizational determinants
Source: PLoS One. 2020 Jun 4;15(6):e0234108. doi: 10.1371/journal.pone.0234108 (PMC7272094; doi:10.1371/journal.pone.0234108)
Supplement: S1 Table — (DOCX) [file pone.0234108.s001.docx]

**S1 Table. Questionnaire.**

| **Variables** | **Items/assumptions** |
| --- | --- |
| **Section 1: Scales and constructs of the proposed model** | |
| Individual variable: Perceived Usefulness | I’m convinced that the EMR will help me carry out my tasks faster |
|  | Using the EMR will greatly improve the effectiveness of my work |
|  | Using the EMR in my work will greatly increase my productivity |
| Individual variable: Perceived Ease of Use | The use of EMR will increase my workload |
|  | Using the EMR I will have more control of my own work |
|  | I will have problems to use the EMR |
|  | I will be able to get the system to do what I want |
|  | The EMR will be easy to use |
| Individual variable: Intention to Use | If I had the opportunity I would use the EMR |
|  | If I had the opportunity I would use the EMR for most of the my work’s processes |
|  | If I had the opportunity I would work in an Hospital where the EMR is already used |
| Normative Factors  (Peer Influence) | The colleagues I value most believe that I should systematically use the EMR |
|  | The colleagues I value most consider the use of EMR as essential for the Hospital |
| Regulative Factors (Adhesion to the Management Objectives) | I very much agree with most of the objectives of the management |
|  | I often come into conflict with the management on the priorities to give to my work |
| **Section 2: Control variables and characteristics of the respondent** | |
| General information  Perceived Risks | Age |
|  | Gender |
|  | Profession |
|  | Clinical Area or Unit |
|  | Academic role in this healthcare company |
|  | Work experience (indicate the number of years) |
|  | Work experience in this healthcare company (indicate the number of years) |
