# Supplementary material for: Electronic Medical Records implementation in hospital: An empirical investigation of individual and organizational determinants
Source: PLoS One. 2020 Jun 4;15(6):e0234108. doi: 10.1371/journal.pone.0234108 (PMC7272094; doi:10.1371/journal.pone.0234108)
Supplement: S2 Table — (DOCX) [file pone.0234108.s002.docx]

**S2 Table. Perceived Usefulness**

|  | | *Totally disagree* | *Strongly disagree* | *Quite disagree* | *Neither agree nor disagree* | *Quite agree* | *Strongly agree* | *Totally agree* | *p-value* |
| --- | --- | --- | --- | --- | --- | --- | --- | --- | --- |
| I’m convinced that the EMR will help me carry out my tasks faster | Nurses | 0 | 0 | 1 | 2 | 32 | 22 | 20 | 0.8 |
|  | Physicians | 0 | 0 | 1 | 2 | 15 | 10 | 7 |  |
| Using the EMR will greatly improve the effectiveness of my work | Nurses | 1 | 0 | 1 | 5 | 32 | 24 | 13 | 0.55 |
|  | Physicians | 0 | 0 | 0 | 6 | 16 | 10 | 3 |  |
| Using the EMR in my work will greatly increase my productivity | Nurses | 1 | 1 | 2 | 15 | 29 | 20 | 9 | 0.43 |
|  | Physicians | 1 | 0 | 1 | 9 | 18 | 4 | 2 |  |
