# Supplementary material for: Electronic Medical Records implementation in hospital: An empirical investigation of individual and organizational determinants
Source: PLoS One. 2020 Jun 4;15(6):e0234108. doi: 10.1371/journal.pone.0234108 (PMC7272094; doi:10.1371/journal.pone.0234108)
Supplement: S3 Table — (DOCX) [file pone.0234108.s003.docx]

**S3 Table. Perceived Ease of Use.**

|  | | *Totally disagree* | *Strongly disagree* | *Quite disagree* | *Neither agree nor disagree* | *Quite agree* | *Strongly agree* | *Totally agree* | *p-value* |
| --- | --- | --- | --- | --- | --- | --- | --- | --- | --- |
| The use of EMR will increase my workload | Nurses | 18 | 12 | 18 | 11 | 9 | 3 | 2 | 0.81 |
|  | Physicians | 2 | 6 | 6 | 8 | 6 | 6 | 0 |  |
| Using the EMR I will have more control of my own work | Nurses | 1 | 1 | 2 | 8 | 31 | 17 | 11 | 0.88 |
|  | Physicians | 1 | 0 | 0 | 5 | 13 | 7 | 8 |  |
| I will have problems to use the EMR | Nurses | 12 | 12 | 19 | 14 | 14 | 0 | 0 | 0.26 |
|  | Physicians | 2 | 8 | 13 | 7 | 3 | 1 | 0 |  |
| I will be able to get the system to do what I want | Nurses | 2 | 8 | 19 | 11 | 16 | 9 | 2 | 0.20 |
|  | Physicians | 1 | 4 | 9 | 11 | 9 | 1 | 0 |  |
| The EMR will be easy to use | Nurses | 0 | 1 | 3 | 10 | 30 | 12 | 8 | **0.019** |
|  | Physicians | 0 | 1 | 5 | 10 | 15 | 2 | 0 |  |
