# Supplementary material for: Electronic Medical Records implementation in hospital: An empirical investigation of individual and organizational determinants
Source: PLoS One. 2020 Jun 4;15(6):e0234108. doi: 10.1371/journal.pone.0234108 (PMC7272094; doi:10.1371/journal.pone.0234108)
Supplement: S4 Table — (DOCX) [file pone.0234108.s004.docx]

**S4 Table. Intention to Use.**

|  | | *Totally disagree* | *Strongly disagree* | *Quite disagree* | *Neither agree nor disagree* | *Quite agree* | *Strongly agree* | *Totally agree* | *p-value* |
| --- | --- | --- | --- | --- | --- | --- | --- | --- | --- |
| If I had the opportunity I would use the EMR | Nurses | 0 | 0 | 0 | 3 | 24 | 18 | 32 | 0.69 |
|  | Physicians | 0 | 0 | 0 | 3 | 9 | 16 | 8 |  |
| If I had the opportunity I would use the EMR for most of the my work’s processes | Nurses | 0 | 0 | 1 | 3 | 30 | 15 | 28 | **0.01** |
|  | Physicians | 0 | 0 | 1 | 3 | 9 | 17 | 6 |  |
| If I had the opportunity I would work in an Hospital where the EMR is already used | Nurses | 2 | 1 | 3 | 14 | 22 | 14 | 18 | 0.31 |
|  | Physicians | 0 | 2 | 1 | 5 | 9 | 13 | 6 |  |
