# Supplementary material for: Electronic Medical Records implementation in hospital: An empirical investigation of individual and organizational determinants
Source: PLoS One. 2020 Jun 4;15(6):e0234108. doi: 10.1371/journal.pone.0234108 (PMC7272094; doi:10.1371/journal.pone.0234108)
Supplement: S5 Table — (DOCX) [file pone.0234108.s005.docx]

**S5 Table. Normative Factors** (Peer Influence).

|  | | *Totally disagree* | *Strongly disagree* | *Quite disagree* | *Neither agree nor disagree* | *Quite agree* | *Strongly agree* | *Totally agree* | *p-value* |
| --- | --- | --- | --- | --- | --- | --- | --- | --- | --- |
| The colleagues I value most believe that I should systematically use the EMR | Nurses | 3 | 3 | 2 | 35 | 10 | 9 | 3 | 0.48 |
|  | Physicians | 0 | 0 | 2 | 17 | 8 | 2 | 3 |  |
| The colleagues I value most consider the use of EMR as essential for the Hospital | Nurses | 2 | 1 | 0 | 23 | 24 | 7 | 8 | 0.54 |
|  | Physicians | 0 | 0 | 2 | 12 | 13 | 3 | 2 |  |
