# Supplementary material for: Electronic Medical Records implementation in hospital: An empirical investigation of individual and organizational determinants
Source: PLoS One. 2020 Jun 4;15(6):e0234108. doi: 10.1371/journal.pone.0234108 (PMC7272094; doi:10.1371/journal.pone.0234108)
Supplement: S6 Table — (DOCX) [file pone.0234108.s006.docx]

**S6 Table. Regulative Factors (Adhesion to the Management Objectives).**

|  | | *Totally disagree* | *Strongly disagree* | *Quite disagree* | *Neither agree nor disagree* | *Quite agree* | *Strongly agree* | *Totally agree* | *p-value* |
| --- | --- | --- | --- | --- | --- | --- | --- | --- | --- |
| I very much agree with most of the objectives of the Management | Nurses | 0 | 1 | 3 | 6 | 27 | 29 | 10 | 0.89 |
|  | Physicians | 1 | 0 | 2 | 3 | 14 | 11 | 4 |  |
| I often come into conflict with the management on the priorities to give to my work | Nurses | 17 | 18 | 17 | 18 | 4 | 3 | 0 | 0.07 |
|  | Physicians | 5 | 4 | 10 | 8 | 8 | 0 | 0 |  |
